# FOCUS: a feature optimization and cascaded unified screening strategy for computer-aided pathology-based glioma molecular subtype classification

**DOI:** 10.1186/s13000-026-01836-1

**Published:** 2026-08-08

**Authors:** Ya Su, Fengqi Li, Hongwei Yang, Jie Bai, Jie Lu

**Affiliations:** 1 Department of Radiology and Nuclear Medicine, Xuanwu Hospital, Capital Medical University, Beijing, 100053 China; 2 Beijing Key Laboratory of Magnetic Resonance Imaging and Brain Informatics, Capital Medical University, No. 45 Changchun Street, Xicheng District, Beijing, 100053 China; 3 Beijing United Imaging Research Institute of Intelligent Imaging, Beijing, China

**Keywords:** IDH mutation status classification, Vision transformer, Patch-based graph convolutional network, N-cadherin-stained pathology slides, Glioma classification

## Abstract

**Background:**

Accurate classification of Isocitrate Dehydrogenase (IDH) mutation status is crucial for the diagnosis and treatment of adult-type diffuse gliomas as defined by the 2021 World Health Organization (WHO) Classification. However, conventional histological and molecular diagnostics are time-consuming, labor-intensive, costly, and often limited in accessibility. Recent advances in artificial intelligence have enabled automated and unbiased glioma classification directly from histopathological images, but challenges remain in capturing complex pathological and molecular features effectively.

**Methods:**

In this study, we propose FOCUS (Feature Optimization and Cascaded Unified Screening), an integrated framework for IDH mutation status classification in gliomas using glioma pathology slides. The histopathological images are divided into patches, from which a Vision Transformer (ViT) extracts interpretable features related to nuclear morphology and tumor progression–associated protein expression. A Patch-based Graph Convolutional Network (PatchGCN) further aggregates spatial context to capture both local morphology and global tumor heterogeneity. A cascaded feature selection strategy is employed to refine biomarker identification, followed by a machine learning classifier for final classification.

**Results:**

Using the proposed framework, the Support Vector Machine (SVM) demonstrated promising performance on a dataset of 73 patients (accuracy = 0.902, AUC = 0.909).

**Conclusions:**

These results highlight the potential of the FOCUS framework as a robust and interpretable tool for IDH mutation status classification, providing a computational approach to distinguish IDH-mutant (astrocytoma and oligodendroglioma) from IDH-wildtype (glioblastoma) tumors.

**Supplementary Information:**

The online version contains supplementary material available at https://doi.org/10.1186/s13000-026-01836-1.

## Introduction

Gliomas constitute the most aggressive and predominant primary malignant brain tumors in adults, comprising approximately 80% of primary central nervous system malignancies [1, 2]. Defined by pronounced inter- and intra-tumoral heterogeneity, these neoplasms exhibit diverse proliferation indices and distinct biological behaviors, which are directly correlated with clinical outcomes [3]. The fifth edition of the World Health Organization Classification of Tumors of the Central Nervous System (WHO CNS5), published in 2021, classifies adult-type diffuse gliomas into three molecularly and prognostically distinct types: (1) astrocytoma, Isocitrate Dehydrogenase (IDH)-mutant; (2) oligodendroglioma, IDH-mutant and 1p/19q-codeleted; (3) glioblastoma, IDH-wildtype [4]. These entities exhibit distinct prognostic and therapeutic profiles. Uniformly, IDH-wildtype glioblastoma is associated with poorer outcomes than IDH-mutant gliomas (comprising astrocytoma and oligodendroglioma), with a median survival of less than two years [5, 6]. Accordingly, determination of IDH mutation status serves as the essential initial stratification step in the diagnostic workflow, separating IDH-mutant from IDH-wildtype tumors and thereby guiding subsequent clinical management.

Glioma diagnosis in current clinical practice involves an integrated assessment of histopathological and molecular features, which remains time-consuming and labor-intensive [7–10]. On one hand, it necessitates neuropathologists to manually examine extensive tissue regions across multiple microscopic resolutions [11–13], and this process is inherently constrained by interobserver variability and the subjective interpretation of ambiguous morphological characteristics [14–17], which directly undermines the accuracy of prognostic stratification; on the other hand, in resource-constrained settings, the availability of histopathological and molecular diagnostic tools is limited, and many facilities lack the infrastructure to perform these essential tests. These limitations underscore the imperative need for more objective, standardized, and widely accessible diagnostic strategies to improve the efficiency and accuracy of glioma classification.

Computer-aided pathology has shown promising efficacy in glioma diagnosis [18–22], with artificial intelligence driving significant recent advancements in analytical efficiency and accuracy [3, 23–32]. In particular, deep learning architectures such as DNNs and ResNet have shown impressive capabilities in extracting histopathological and molecular features, achieving notable success in classifying IDH mutation status [9, 33–36]. Moreover, deep transfer learning-based approaches have further improved classification performance, thereby enhancing diagnostic accuracy [37, 38]. However, current methods still face limitations in feature extraction, interpretability, and predictive accuracy, constraining clinical applicability.

In this work, we propose a novel Feature Optimization and Cascaded Unified Screening (FOCUS) strategy for IDH mutation status classification based on glioma pathology slides. The proposed strategy first leverages a Vision Transformer (ViT) [39] to extract representative features from patches of pathological images. To preserve the local structural relationships and spatial dependencies within the tissue architecture, Patch-based Graph Convolutional Network (PatchGCN) is incorporated [40–42]. A tailored feature selection mechanism is then applied to refine the extracted features. Finally, a machine learning classifier is utilized for classification. Our approach addresses the limitations in histopathological feature extraction and provides a systematic and interpretable framework for glioma classification.

## Materials and methods

### Dataset curation

This retrospective cohort study included 90 patients with histologically confirmed adult-type diffuse glioma treated at Xuanwu Hospital, Capital Medical University, between 2022 and 2023. The following cases were excluded: 4 patients with incomplete clinicopathological data, 8 patients lacking IDH mutation and 1p/19q codeletion status, and 5 patients with secondary glioblastoma. The final cohort consisted of 73 patients (aged 23–81 years, mean 50 ± 16 years; male: 41, female: 32), categorized into two molecular subgroups according to IDH mutation status, aligning with the 2021 WHO classification framework: Group 0 (IDH-mutant, *n* = 31; comprising both astrocytoma and oligodendroglioma) and Group 1 (IDH-wildtype, *n* = 42; corresponding to glioblastoma, IDH-wildtype).

Formalin-fixed, paraffin-embedded (FFPE) tumor tissues from enrolled patients were used for immunohistochemical (IHC) detection of N-cadherin (NCAD), a calcium-dependent adhesion molecule associated with glioma subtypes and invasiveness [43, 44]. Sections were counterstained with hematoxylin to visualize nuclear morphology. The NCAD-stained slides were scanned for computational extraction of glioma pathological features. In parallel, IDH1 and IDH2 mutational hotspots were analyzed by Sanger sequencing using DNA from the corresponding FFPE samples. Detailed protocols are provided in the Supplementary Materials. All NCAD-stained pathology slides underwent quality assessment by two senior neuropathologists with over 6 years of diagnostic experience. For each case, the slide with the highest staining quality was selected for subsequent image acquisition. This study was approved by the Ethics Committee of Xuanwu Hospital Capital Medical University (No. 2023-044). Informed consent was waived due to the retrospective analysis.

### Integrated FOCUS strategy for glioma classification

**Fig. 1:**
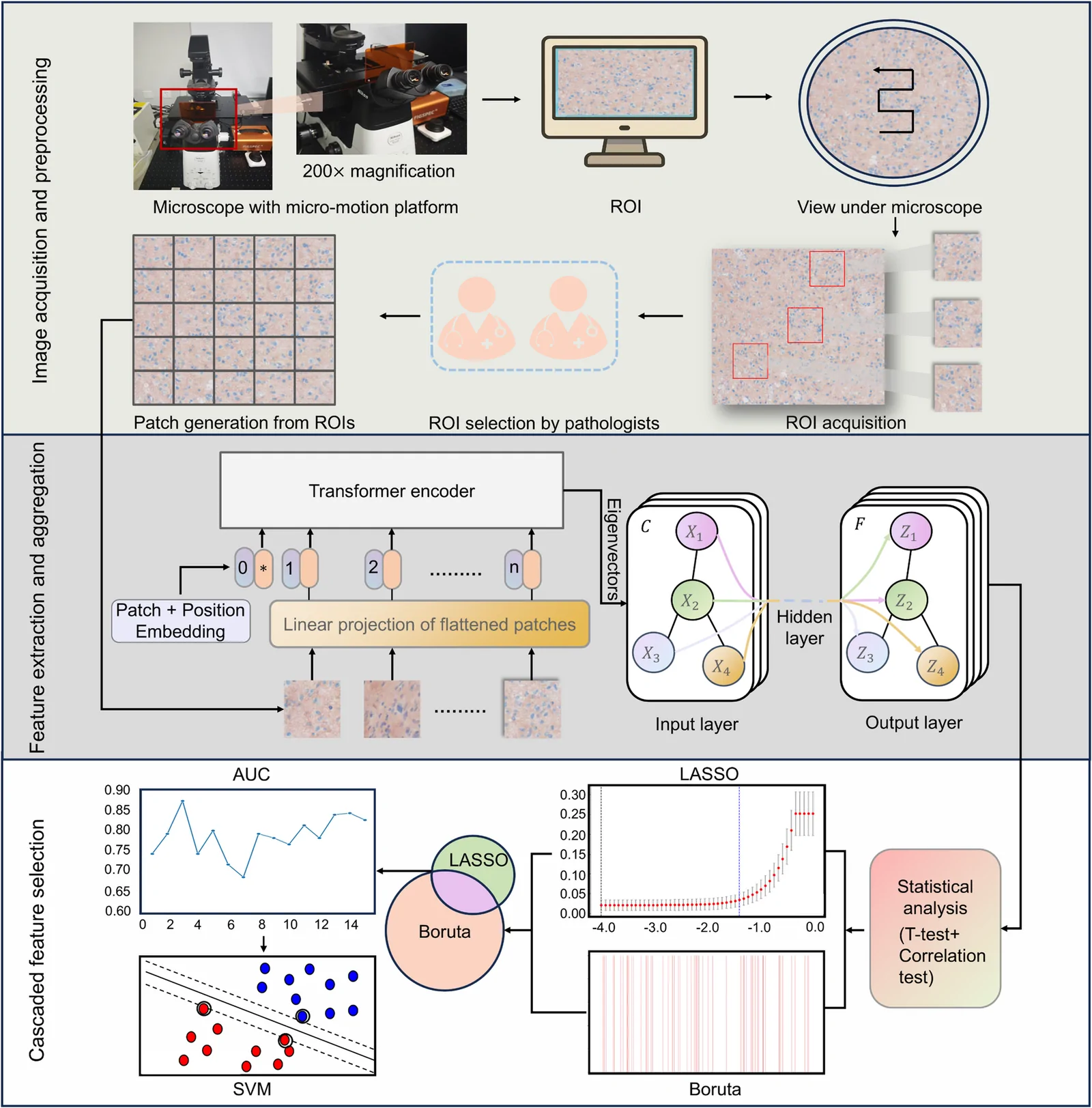
The workflow of this study. Glioma pathology slide images are scanned at 200× magnification and partitioned into non-overlapping patches. Each patch is processed by a Vision Transformer (ViT) to extract global contextual features. A Patch-level Graph Convolutional Network (PatchGCN) is subsequently applied to model spatial relationships among patches. The extracted features are then subjected to a cascaded selection process involving statistical analysis, hybrid least absolute shrinkage and selection operator (LASSO)-boruta feature selection, and area under the curve (AUC)-based screening. The most discriminative features are finally input into a support vector machine (SVM) classifier to achieve robust IDH mutation status prediction

Figure 1 illustrates the workflow of the proposed FOCUS strategy, which integrates ViT for initial feature extraction and PatchGCN for spatial feature aggregation, followed by cascaded feature selection and final classification using a machine learning classifier (e.g., Support Vector Machine (SVM)). In this framework, images of glioma pathology slides are acquired using a microscope with a micro-motion platform at 200× magnification. The acquired images are subsequently divided into non-overlapping patches. Each patch is processed by a ViT to capture global contextual information. PatchGCN is then employed to aggregate spatial relationships among patches, enhancing local structural representations while also capturing global tumor heterogeneity. The resulting features undergo a cascaded selection process involving statistical analysis, hybrid least absolute shrinkage and selection operator (LASSO)-boruta feature selection, and area under the curve (AUC)-based screening to retain the most discriminative histological and molecular features. Finally, the selected features are fed into a machine learning classifier for robust IDH mutation status prediction.

### Image acquisition and preprocessing

Initially, a programmable microscope platform was employed to randomly capture 15 regions of interest (ROIs) per case at 200× magnification, with each ROI measuring 534 × 480 pixels. Subsequently, two board-certified pathologists independently and blindly evaluated these ROIs, applying inclusion criteria of ≥ 80% tumor cellularity while excluding specimens containing extraneous background artifacts or normal cellular constituents.

All qualified ROIs underwent standardized preprocessing to ensure consistent feature extraction across heterogeneous histopathological images. First, Macenko normalization [45] was implemented to address staining discrepancies between distinct slides. Pixel intensities were then scaled to the [0, 1] range, followed by Z-score standardization to normalize feature magnitudes. Spatial cropping was performed to divide each ROI into 100 × 100-pixel analytical patches, yielding a total of 4,915 patches for Group 0 and 5,202 patches for Group 1.

All experiments were conducted on a high-performance computing workstation equipped with an NVIDIA RTX 4090 GPU, an Intel Core i9-13900 CPU, and 128 GB of RAM. The dataset was split into approximately 70% training and 30% testing at the patient level, with all patches from each patient assigned exclusively to one set. This prevents data leakage and ensures generalizability. All preprocessing adhered to standardized protocols to ensure data consistency and reproducibility for downstream analysis.

### Feature extraction and aggregation

ViT architecturally processes the patches through hierarchical integration of Multi-Head Self-Attention mechanisms to discern high-dimensional feature representations. Each analytical patch is first projected into an n-dimensional latent space via a learnable embedding layer, after which global contextual relationships are captured through the transformer’s self-attention mechanism. This computational paradigm enables the model to dynamically attend to nuclear morphological and progression-associated histopathological features within glioma microenvironments, while explicitly preserving non-local spatial dependencies that conventional convolutional neural networks often fail to capture due to their inherent inductive biases.

Following patch-level feature extraction, PatchGCN utilizes graph convolutional mechanisms to synthesize multi-scale contextual embeddings by aggregating cross-patch information at the patient level. Through adaptive graph construction, it establishes topological dependencies among histopathological units, enabling holistic feature integration while preserving localized discriminative patterns intrinsic to individual patches. The hierarchical processing pipeline systematically encodes each case’s diagnostic information into a compact n-dimensional manifold representation, which serves as discriminative feature vectors for downstream classification tasks.

### Cascaded feature selection

#### Statistical analysis

This study implements a tiered feature selection framework employing a progressive refinement strategy to iteratively eliminate collinear biomarkers while preserving maximally discriminative molecular signatures for glioma classification. Statistical filtering was performed using Python (version 3.9.2) with SciPy 1.7.3’s statistical modules, executing two-tailed independent Student’s t-tests under validated assumptions of normality and homoscedasticity (α = 0.05). Features exhibiting statistically significant differential expression (*p* < 0.05) between IDH-mutant (Group 0) and IDH-wildtype (Group 1) subgroups were systematically retained. Through this rigorous multivariate analysis pipeline, 572 non-redundant biomarkers were ultimately selected for subsequent classification modeling.

Redundant features are further eliminated through correlation testing. First, the Shapiro-Wilk normality test is performed for each feature. For normally distributed features, the Pearson correlation coefficient is used, while the Spearman rank correlation coefficient is applied to non-normally distributed features. The correlation matrix of all features was computed, and its corresponding heatmap is presented in Supplementary Figure S1. A threshold of |r| > 0.8 is set. If a feature pair exceeds this threshold, the feature with the lower correlation to the target variable is deleted. To further refine the selection, K-means clustering (with 3 clusters) is applied to group features, retaining the feature with the highest correlation within each cluster. Ultimately, 411 features are retained. See Supplementary Material for more details about the correlation test.

#### Hybrid LASSO-boruta feature selection

We employed L1 regularized LASSO logistic regression to select the most informative features for glioma classification. LASSO improves both predictive performance and model interpretability by applying an L1 penalty on the absolute values of regression coefficients. This penalty encourages sparsity by shrinking the coefficients of less informative features toward zero, thereby enabling automatic variable selection. The method is particularly well-suited for high-dimensional datasets with multicollinearity [46–48]. The level of sparsity is governed by the regularization parameter λ, which determines the strength of the L1 penalty. A larger λ value applies stronger regularization, resulting in more coefficients being reduced to zero and simplifying the model. To identify a suitable balance between model simplicity and predictive accuracy, we conducted tenfold cross-validation over a logarithmically spaced λ range from 10⁻⁴ to 10⁰. The λ parameter was chosen using the one-standard-error approach, prioritizing a simpler model with a validation error within one standard error of the lowest observed. This approach reduces the risk of overfitting while producing a more compact and generalizable set of features.

Boruta is a feature selection algorithm based on a random forest classifier. Unlike general feature selection methods, which aim to identify a minimal, compact feature set optimal for a specific model, boruta focuses on selecting features most relevant to the dependent variable [49]. We use boruta for screening by generating ‘shadow features’ through random permutation of the original features, thus constructing an extended feature set. A random forest classifier (500 trees, Gini impurity criterion) is trained on this set, and the original features are compared with their corresponding shadow features over multiple iterations. If a feature’s importance consistently and significantly exceeds that of its shadow feature (one-tailed Z-test, *p* < 0.01), it is considered statistically significant.

LASSO regression and the boruta algorithm are integrated to leverage their complementary strengths in linear sparsity and nonlinear importance evaluation in this work. Features selected by both LASSO and boruta are prioritized. This hybrid strategy aims to balance LASSO’s sparsity efficiency with boruta’s nonlinear discriminative power, ensuring comprehensive coverage of both linear effects and interaction terms [50].

#### AUC-based screening

To optimize the feature set and maximize model performance, the study implements an AUC-based feature screening strategy. First, all features undergo Z-score standardization to eliminate scale differences in feature importance evaluation. A random forest model is trained with the training data to compute feature importance scores. Features are ranked in descending order of importance to form an ordered sequence. Forward stepwise feature selection is then performed, starting with the highest-ranked feature and progressively incorporating secondary features based on importance. This process continues until all candidate features are included. By analyzing the trend of AUC values relative to the number of features (Supplementary Figure S2), the optimal feature combination that yields peak is determined. In this study, the AUC reaches its peak when the number of features reaches three, and these three features are retained. See Supplementary Material for more details about the AUC-based screening.

### Model development and performance evaluation

Seven binary classification models were implemented and compared for IDH mutation status prediction: SVM, Stochastic Gradient Descent (SGD), K-Nearest Neighbors (KNN), Random Forest, eXtreme Gradient Boosting (XGBoost), Light Gradient Boosting Machine (LightGBM), and Tabular Prior-Data Fitted Network (TabPFN). All models were implemented using Python (version 3.9.2) with scikit-learn (version 1.0.2), XGBoost (1.5.0), LightGBM (3.3.2), and TabPFN (0.1.9) libraries.

The dataset was split at the patient level into a training set (70%) and an independent test set (30%). Within the training set, we performed 10 repeats of 5-fold cross-validation to select optimal hyperparameters for each classifier. Specifically, the training data were randomly partitioned into five folds; four folds were used for training and one for validation, repeated five times to cover all folds. This process was repeated 10 times with different random splits. The hyperparameter combination yielding the highest average validation AUC across all repeats was selected. The final model was then retrained on the entire training set using the optimal hyperparameters and evaluated on the held-out test set. This nested cross-validation design ensures unbiased performance estimation and mitigates overfitting.

The performance of the model in this experiment was primarily evaluated using the following metrics: receiver operating characteristics (ROC) curves, decision curve analysis (DCA), as well as accuracy, precision, recall, and F1-score. To account for the uncertainty arising from the limited test set size, we computed 95% confidence intervals (CIs) for all metrics. For AUC, we used DeLong’s non-parametric method to estimate the 95% CI. This method is specifically designed for AUC and provides an asymptotically valid interval based on the theory of U-statistics. For accuracy, precision, recall, and F1-score, we applied bootstrap resampling with 2,000 iterations. In each iteration, the test set was resampled with replacement to the same size, and the metric was recalculated. The 2.5th and 97.5th percentiles of the 2,000 bootstrap estimates were taken as the 95% CI.

To assess whether the observed differences in AUC between models were statistically significant, we conducted pairwise DeLong tests for all model combinations. A p-value < 0.05 was considered statistically significant.

## Results

### Overview and patient characteristics

A total of 73 histologically confirmed glioma patients from Xuanwu Hospital were included in this study (mean age, 50 years ± 16 [standard deviation]; 41 male and 32 female patients), including 31 IDH-mutant gliomas and 42 IDH-wildtype gliomas. Pathological images were obtained from NCAD-immunostained and hematoxy-lin-counterstained tissue sections of these patients, emphasizing detailed nuclear morphology. Following standardized preprocessing, the images were segmented into analytical patches of 100 × 100 pixels. Details of the dataset and preprocessing procedures are provided in "Dataset curation" and "Image acquisition and preprocessing" section. Ultimately, 4,915 IDH-mutant and 5,202 IDH-wildtype patches were obtained for analysis.

### Feature extraction and aggregation

**Fig. 2:**
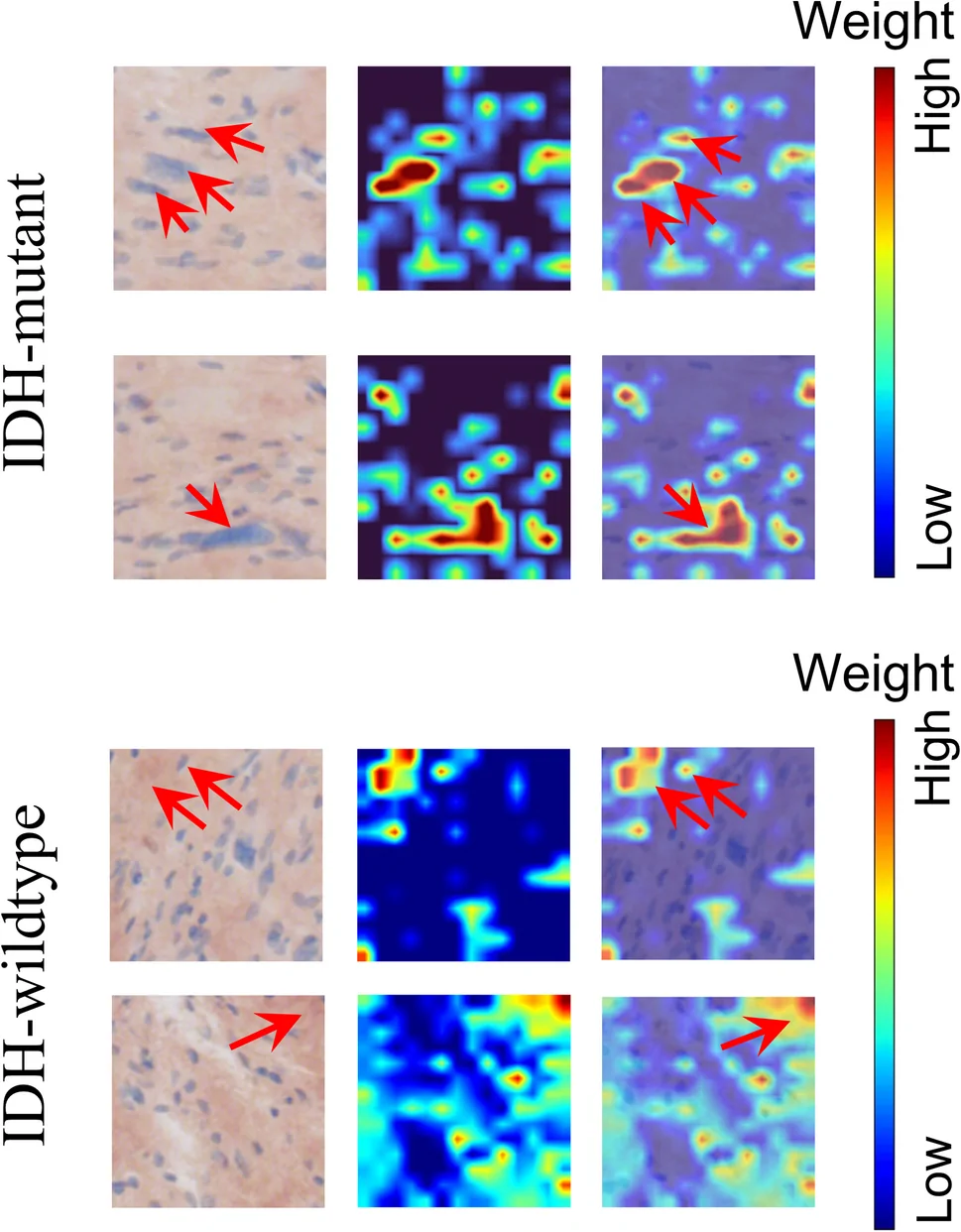
Comparative visualization of ViT-based spatial attention across IDH-mutant and IDH-wildtype glioma patches. The gradient colors represent attention weights assigned by the model, with warmer colors indicating higher attention. From left to right: original histopathological patch image, ViT-derived attention heatmap, and the merged overlay. Red arrows mark representative high-attention regions identified by the model, corresponding to nuclear morphology and intensely brown-stained areas indicative of elevated NCAD expression

The patches were first input into ViT, where each patch was projected into a 768-dimensional feature vector through linear transformation and multi-head self-attention mechanisms. These patch-level feature representations captured both local morphological details and global contextual dependencies within the images of NCAD-stained slides with hematoxylin counterstain. The visualization results of feature extraction in Fig. 2 illustrate the spatial attention distribution across the patches. Using gradient-weighted class activation mapping, the generated heatmaps highlight regions where the model assigns high attention, emphasizing nuclear pleomorphism and tumor-relevant areas with high NCAD expression. The ViT model effectively captures long-range dependencies and morphological patterns, focusing on key regions that contribute to glioma classification. The color intensity in the heatmaps reflects the model’s confidence in specific tissue structures, with warmer regions indicating stronger feature activation. By overlaying the heatmap onto the original image, nuclei and areas with deeper brown staining are prominently highlighted, demonstrating a strong correlation between the model’s focus and pathologist-annotated malignant regions, thus underscoring the interpretability of the model’s feature extraction.

Following patch-level feature extraction, PatchGCN was employed to further refine and aggregate the 768-dimensional feature vectors across all patches within a given sample. By constructing a graph structure based on spatial adjacency, PatchGCN propagated and integrated information between patches, enhancing feature robustness while preserving spatial relationships. After feature aggregation, the model ultimately produced a 768-dimensional feature vector per sample, ensuring a compact yet informative representation for subsequent classification tasks.

### Hybrid LASSO-boruta feature selection

**Fig. 3:**
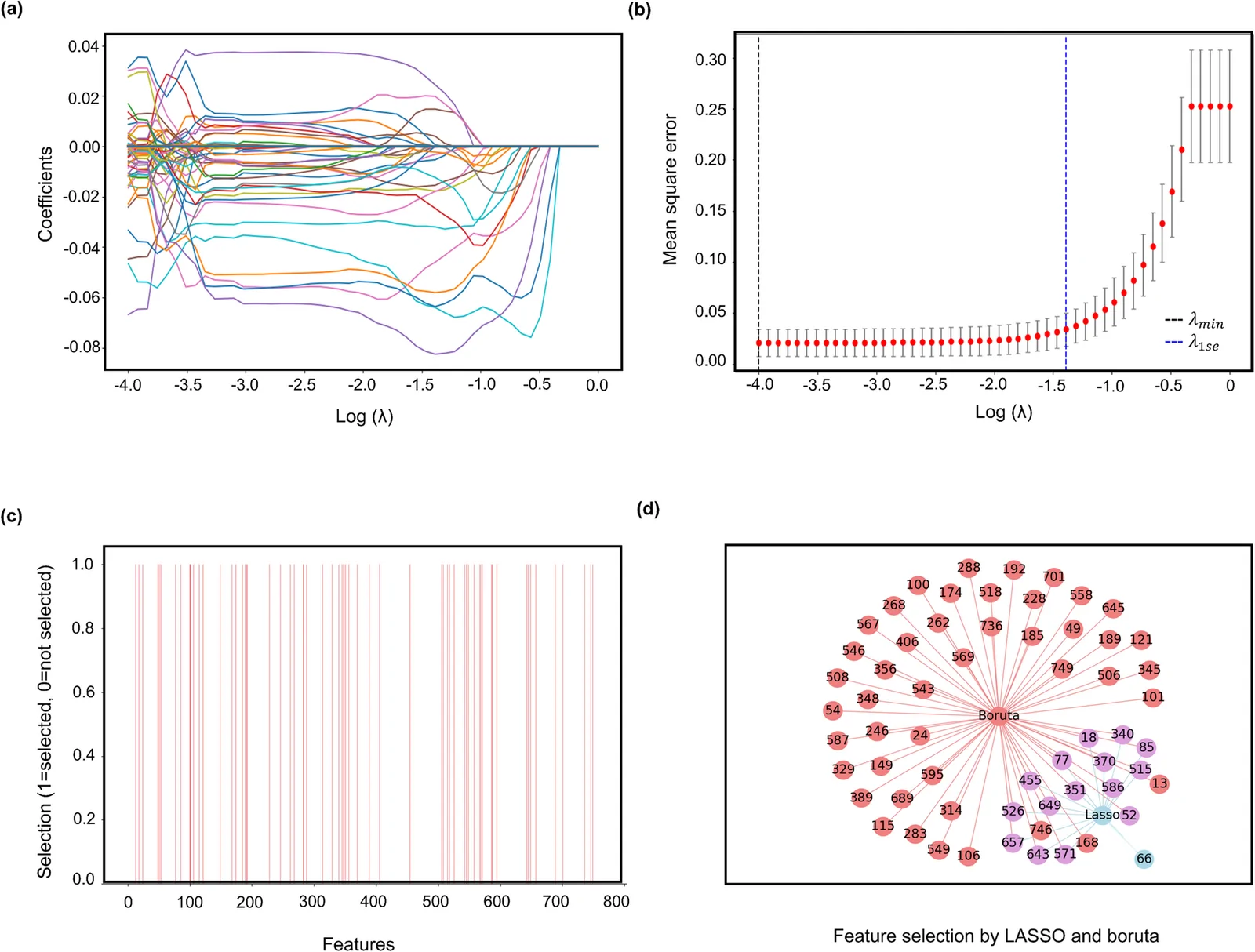
LASSO and boruta hybrid features selection. **a** Coefficient trajectories of individual features under different λ, each curve represents the coefficient trajectory of a single feature. **b** Cross-validation curve for LASSO regression showing mean squared error (MSE) as a function of log(λ). Error bars represent ± 1 standard error. The two vertical dashed lines mark λ_min_ (the value of λ with the minimum MSE) and λ_1se_ (the largest λ such that the MSE is within one standard error of the minimum), respectively. **c** Boruta feature selection diagram. **d** Common predictors between LASSO and boruta

Following feature selection via t-tests and correlation analysis, the remaining features were subjected to a joint selection process using LASSO regression and the boruta algorithm. Figure 3a illustrates the variation of feature coefficients under different regularization parameters λ in the LASSO regression. With λ increasing, all coefficients tend toward zero due to strong regularization; as λ decreases, the weights of informative features gradually emerge, while non-informative features remain at zero, highlighting the feature selection effect.

The optimal λ value was determined via 10-fold cross-validation. Figure 3b presents the curve of mean squared error (MSE) across different λ values. The left dashed line indicates λmin, the regularization strength that yields the lowest MSE, while the right dashed line denotes λ1se, the most parsimonious model whose MSE lies within one standard error of the minimum. To achieve a better balance between model complexity and generalization performance, λ1se (0.041) was selected as the final regularization parameter, resulting in the retention of 16 non-zero coefficient features by the LASSO model.

The boruta algorithm identifies features consistently exhibiting significantly higher importance as “confirmed important.” through iterative comparisons of importance scores between real and shadow features. As shown in Fig. 3c, each red vertical bar represents a feature confirmed by boruta. A total of 60 features were selected as potentially informative.

To leverage the complementary strengths of both methods, we computed the intersection of features retained by LASSO and boruta. Ultimately, 15 features were simultaneously identified as important by both algorithms and preserved for downstream analysis (Fig. 3d). This joint selection strategy combines the sparsity and efficiency of LASSO with the global stability of boruta’s importance evaluation, effectively improving the robustness of the selected feature set and enhancing the generalization capability of the model.

### Comparisons of model performance

**Fig. 4:**
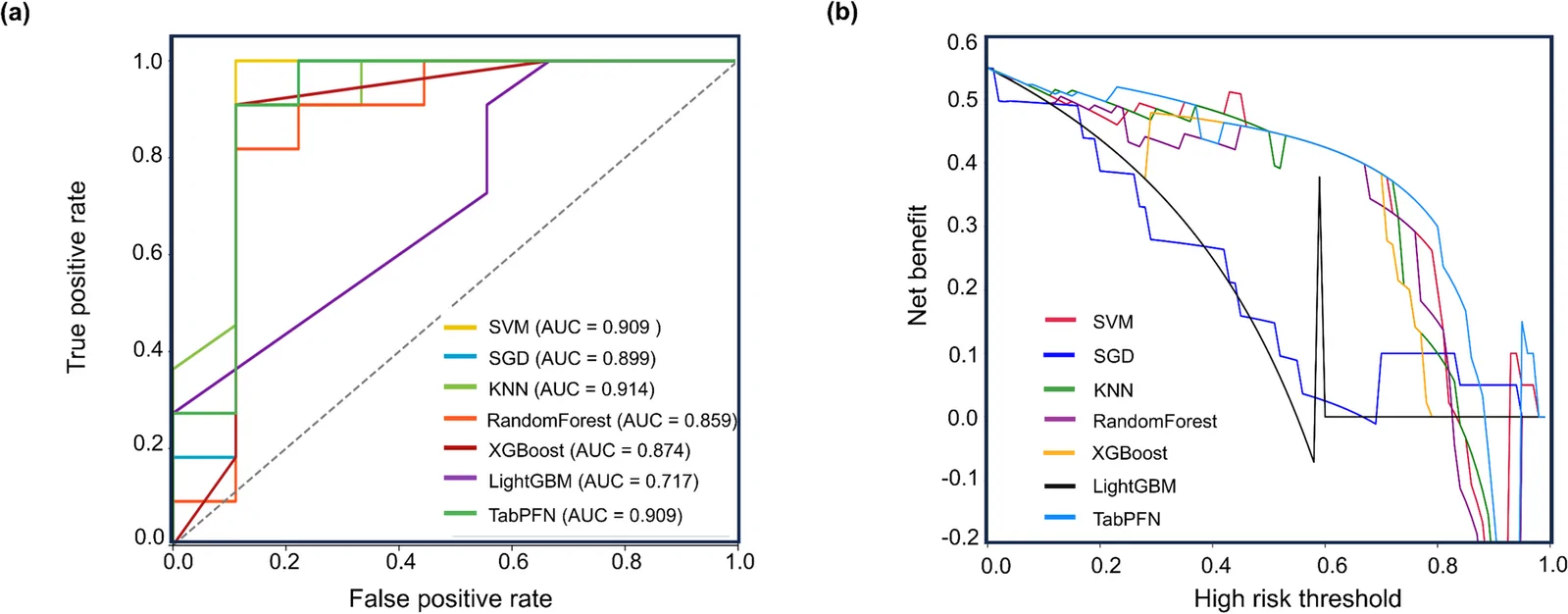
Comparative performance evaluation of classification models. **a **Receiver operating characteristics (ROC) Curve analysis for seven machine learning models (SGD, KNN, RandomForest, eXtreme Gradient Boosting (XGBoost), Light Gradient Boosting Machine (LightGBM), Tabular Prior-Data Fitted Network (TabPFN), and Proposed Model) with 95% confidence intervals (shaded regions); (**b**) Decision curve analysis (DCA) of clinical net benefit versus high-risk threshold probability (0–1.0) across seven models

Seven binary classification models — SGD, KNN, RandomForest, XGBoost, LightGBM, TabPFN, and SVM — were evaluated on the feature-screened dataset for glioma IDH mutation status classification. Each model was trained and validated using five-fold cross-validation. As shown in Fig. 4a, all models except LightGBM (AUC = 0.717) demonstrated strong discriminative capabilities for IDH mutation status classification, with AUC values exceeding 0.850. Among them, KNN achieved the highest AUC (0.914), followed closely by SVM (0.909) and TabPFN (0.909).

DCA was performed to evaluate the clinical utility of the seven classification models across a range of threshold probabilities (Fig. 4b). In the threshold range of 0–0.2, all models exhibited similar net benefit. In the threshold range of 0.2–0.6, multiple models demonstrated competitive performance: TabPFN and SVM frequently achieved the highest net benefit, followed closely by KNN and XGBoost. For thresholds exceeding 0.6, TabPFN and SVM continued to yield relatively favorable net benefit up to approximately 0.8, beyond which all models showed declining and eventually negative net benefit. In contrast, SGD and LightGBM consistently underperformed across most thresholds.

Furthermore, the models were compared using accuracy, precision, recall, and F1-score metrics, as presented in (Table 1). Overall accuracy ranged from 0.602 (LightGBM) to 0.902 (SVM). Three models achieved accuracy above 0.8: SVM (0.902), TabPFN (0.852), and RandomForest (0.803), with SVM being the only model exceeding 0.9. SVM achieved the highest accuracy (0.902), along with precision of 0.911, recall of 0.906, and an F1-score of 0.908. TabPFN, as the second-best in accuracy (0.852), demonstrated a recall of 0.907, precision of 0.836, and F1-score of 0.870.

**Table 1:** Performance evaluation of models

Model	Accuracy (95% CI)	Precision (95% CI)	Recall (95% CI)	F1-Score (95% CI)	AUC (95% CI)
SVM	0.902 [0.753, 1.000]	0.911 [0.714, 1.000]	0.906 [0.714, 1.000]	0.908 [0.750, 1.000]	0.909 [0.729, 1.000]
SGD	0.692 [0.485, 0.879]	0.791 [0.333, 1.000]	0.663 [0.391, 0.992]	0.721 [0.374, 0.989]	0.899 [0.719, 1.000]
KNN	0.795 [0.598, 0.942]	0.763 [0.527, 1.000]	0.912 [0.717, 1.000]	0.830 [0.632, 0.966]	0.914 [0.783, 1.000]
RandomForest	0.803 [0.603, 0.954]	0.814 [0.551, 1.000]	0.824 [0.551, 1.000]	0.819 [0.588, 0.960]	0.859 [0.655, 1.000]
XGBoost	0.753 [0.600, 0.905]	0.896 [0.663, 1.000]	0.816 [0.554, 1.000]	0.854 [0.664, 1.000]	0.874 [0.693, 1.000]
LightGBM	0.602 [0.401, 0.800]	0.618 [0.337, 0.879]	0.725 [0.442, 1.000]	0.666 [0.400, 0.857]	0.717 [0.474, 0.960]
TabPFN	0.852 [0.707, 1.000]	0.836 [0.600, 1.000]	0.907 [0.712, 1.000]	0.870 [0.667, 1.000]	0.909 [0.747, 1.000]

To determine whether the observed differences in AUC among models were statistically significant, we performed pairwise DeLong tests (Supplementary Table S1). All comparisons yielded p-values > 0.05, indicating that no model demonstrated statistically superior discriminative ability over another in terms of AUC. 

Nevertheless, SVM exhibited the highest point estimates for accuracy (0.902), precision (0.911) and F1-score (0.908), along with competitive AUC (0.909) and favorable decision curve performance across clinically relevant threshold ranges. While KNN achieved the highest AUC point estimate (0.914), its advantage over SVM was marginal (Δ = 0.005). TabPFN, although competitive in AUC (0.909) and recall (0.907), yielded lower accuracy (0.852) and precision (0.836) than SVM.

Considering the lack of statistical significance in AUC comparisons (all *p* > 0.05; Supplementary Table S1) and the limited sample size of this study, we interpret SVM as offering a potentially favorable balance of performance metrics within this dataset. However, these findings should be viewed as preliminary and specific to our cohort; the selection of an optimal model for clinical deployment would require further validation on larger, multi-institutional datasets.

The predictive probabilities of different models for both IDH-mutant and IDH-wildtype gliomas on the testing set are presented in (Table 2). Each row corresponds to a patient sample, with ground truth labels indicating class 0 for IDH-mutant and class 1 for IDH-wildtype. The SVM model demonstrated relatively high consistency with the ground truth, providing high-confidence predictions across most cases. For example, Sample 18, which was labeled as IDH-mutant, was misclassified by several models. The misclassifications may be attributed to the intermediate-level morphological or molecular characteristics of sample 18, which make it difficult to distinguish confidently. In contrast, SVM correctly identified its subtype, suggesting better sensitivity to cases with subtle or borderline features.

**Table 2:** Prediction of Isocitrate Dehydrogenase (IDH) molecular subtype on the test set

Sample ID	Ground truth	SVM	SGD	KNN	RandomForest	XGBoost	LightGBM	TabPFN
1	1	0.976	0.945	0.901	0.917	0.7877	0.594	0.974
2	1	0.851	0.447	0.724	0.835	0.727	0.594	0.913
3	0	0.045	0.001	0.002	0.130	0.287	0.487	0.021
4	0	0.243	0.005	0.146	0.115	0.287	0.394	0.127
5	1	0.791	0.265	0.832	0.761	0.705	0.494	0.859
6	0	0.927	0.697	0.891	0.944	0.787	0.594	0.943
7	1	0.807	0.193	0.921	0.875	0.772	0.614	0.901
8	0	0.233	0.005	0.113	0.125	0.287	0.587	0.153
9	1	0.909	0.558	0.927	0.822	0.771	0.594	0.953
10	0	0.146	0.001	0.119	0.223	0.287	0.387	0.075
11	1	0.451	0.014	0.481	0.248	0.287	0.485	0.378
12	0	0.005	6.833e-06	0.005	0.186	0.287	0.587	0.012
13	0	0.263	0.004	0.291	0.544	0.287	0.587	0.219
14	0	0.344	0.008	0.561	0.277	0.287	0.294	0.225
15	1	0.947	0.837	0.901	0.812	0.787	0.594	0.972
16	1	0.881	0.517	0.739	0.488	0.752	0.594	0.922
17	1	0.820	0.428	0.893	0.948	0.787	0.491	0.886
18	0	0.422	0.036	0.523	0.348	0.287	0.597	0.517
19	1	0.712	0.165	0.756	0.764	0.499	0.587	0.804
20	1	0.813	0.289	0.739	0.830	0.787	0.594	0.892

Sample 6 and 11 were incorrectly predicted by all models. Upon further examination, these samples exhibited weak and non-discriminative feature signals, lacking clear patterns associated with either subtype. This ambiguity may have caused uncertainty in the classification boundaries of all models, resulting in consistent errors. These findings emphasize the limitations of current models in handling feature-deficient or borderline cases and highlight the potential value of integrating additional data modalities or uncertainty-aware learning frameworks to improve robustness.

## Discussion

In this study, we propose an integrated deep learning–based strategy for automated classification of IDH mutation status in gliomas using pathological sections, aiming to facilitate objective and efficient molecular diagnosis. The proposed strategy directly addresses the limitations of conventional histological and molecular diagnostics in clinical practice, potentially improving diagnostic accuracy and patient prognosis.

This framework integrates deep feature learning, spatial aggregation, systematic feature selection, and robust classification into a unified workflow, providing a rigorous and adaptable methodological framework for automated molecular subtyping from pathological slides. ViT and PatchGCN are employed to learn high-level discriminative representations that capture fine-grained morphology and spatial tissue architecture—patterns that are challenging to encode using traditional hand-crafted features. This deep feature extraction constructs a comprehensive feature space that serves as a rich information reservoir for subsequent analysis. The cascaded feature selection pipeline subsequently refines this high-dimensional representation by eliminating redundant and noisy features, thereby mitigating overfitting and enhancing generalizability—a critical consideration given the limited sample size typical of clinical cohorts. Furthermore, the simplicity and interpretability of the resulting model are essential for clinical decision support.

Previous studies have explored diverse computational approaches for glioma classification using pathological images. Rathore et al. [31] employed handcrafted texture descriptors, including the gray-level co-occurrence matrix and gray-level run-length matrix, to characterize histopathological patterns for glioma grading. Im et al. [37] applied deep transfer learning models to classify diffuse glioma subtypes from clinical-grade pathological images. Pei et al. [30] further integrated deep neural network analysis of pathology images with molecular data to improve glioma classification performance. However, traditional texture-based methods inadequately capture complex feature interactions, while deep learning approaches often lack explicit feature selection, reducing interpretability. Our framework addresses this by integrating LASSO and Boruta. This approach synergistically combines the sparsity-inducing efficiency of LASSO with the nonlinear discriminative power of Boruta, thereby ensuring comprehensive coverage of both linear effects and interaction terms. Consequently, our strategy reduces feature redundancy while preserving informative pathological characteristics, enhancing the robustness of downstream classification models.

SVM, KNN, and TabPFN all achieved AUC > 0.900 (Fig. 4a). However, pairwise DeLong tests revealed no statistically significant differences among any models (all *p* > 0.05; Supplementary Table S1), and the wide, overlapping 95% CIs (Table 1) indicate substantial uncertainty in performance rankings—warranting cautious interpretation given the limited sample size. Despite this, SVM attained a favorable balance across multiple metrics. RandomForest (AUC = 0.859) and XGBoost (AUC = 0.874) showed stable performance, while LightGBM lagged (AUC = 0.717). Challenging cases such as Samples 5 and 17 were misclassified by several models, highlighting difficulty with feature-deficient or borderline cases. Importantly, the primary goal of this study was to validate the FOCUS pipeline, not to identify an optimal classifier; further evaluation on larger, multi-center cohorts is needed for real-world deployment. This is especially important given the well-recognized molecular heterogeneity across glioma subtypes [51–53] and the complex biology of IDH-wildtype glioblastoma [54–56].

Decision curve analysis (Fig. 4b) showed that TabPFN excelled at low thresholds (high sensitivity), while SVM and XGBoost offered balanced performance at moderate thresholds. LightGBM yielded consistently lower net benefit, and SGD showed instability. Overall, SVM and TabPFN demonstrated superior reliability in balancing sensitivity and specificity.

In this study, NCAD immunohistochemical staining with hematoxylin counterstaining was adopted instead of conventional hematoxylin and eosin (H&E) staining to enhance the interpretability of pathological features in glioma classification. Conventional H&E slides mainly emphasize nuclear morphology through hematoxylin staining and depict the cytoplasm in pink due to eosin binding to acidophilic proteins and organelles [57, 58].

However, this cytoplasmic signal represents an averaged mixture of cellular components with limited diagnostic specificity. In contrast, NCAD staining labels N-cadherin, a calcium-dependent cell adhesion molecule associated with tumor grade, cell invasiveness, and differentiation [43, 44, 59, 60]. By using NCAD-stained slides, our approach preserves key nuclear morphological and textural information while simultaneously providing molecular-level insight into NCAD expression patterns. This enables precise quantification of NCAD positivity within tissue sections and facilitates a more comprehensive characterization of glioma histopathology. Although NCAD is not an entirely subtype-specific marker, it offers greater biological relevance than the nonspecific cytoplasmic eosin signal in H&E slides. In this sense, the proposed method extends conventional morphological analysis by integrating a protein-level expression dimension, providing a more biologically meaningful representation of tumor heterogeneity. Nevertheless, the extent to which NCAD-based imaging enhances diagnostic accuracy compared with H&E staining remains to be further validated through larger and multi-institutional datasets. Future research should therefore quantify the contribution of NCAD expression to classification improvement. To address this, we are planning a comparative study utilizing paired NCAD and H&E slides from the same tumor specimens collected across seven partner hospitals. This initiative will enable us to quantify the relative performance of both staining approaches. Furthermore, leveraging the distinct physiological information captured by each staining method, we will analyze the interpretable features driving classification performance, thereby providing mechanistic insights into the added diagnostic value of NCAD’s targeted molecular signal over conventional eosinophilic appearance. Overall, the use of NCAD-stained slides in our framework is both rational and innovative, as it bridges nuclear morphology with molecular expression, offering an enriched and interpretable foundation for automated glioma subtype classification.

We envision the FOCUS strategy being deployed as an assistive decision-support tool rather than a fully automated system. In routine practice, whole-slide images would be processed through the feature extraction and selection pipeline, with the classifier output—a probability score for IDH status—presented to pathologists as a “second opinion” to aid in ambiguous cases. The model’s interpretability (attention heatmaps and sparse feature set) facilitates clinical trust. Potential applications include triage in high-volume centers and cost-effective preliminary subtyping in resource-limited settings.

Despite yielding promising outcomes, several limitations persist. First, while the NCAD-based analysis demonstrates feasibility, the framework’s applicability to conventional H&E slides and other immunohistochemical markers remains to be established. Notably, H&E staining remains the clinical standard for glioma histopathology. To address this, the framework is designed with staining adaptability in mind: its feature extraction and selection pipeline is staining-agnostic, allowing retraining or transfer learning on H&E-stained slides. Future work will systematically evaluate this adaptability using paired NCAD and H&E specimens. Second, whether NCAD offers diagnostic advantage over conventional H&E for IDH mutation status classification remains to be determined. Future work will systematically compare both staining approaches and analyze the interpretable features driving performance to elucidate the diagnostic information conferred by each method. Third, the relatively small sample size (73 patients) and the single-center dataset may undermine generalizability due to variations in regional pathological protocols, scanner configurations and patient populations across diverse clinical settings. To address this, we are conducting a multi-center validation study with seven partner hospitals, accumulating a larger and more diverse dataset, which will enable rigorous evaluation of model robustness and generalizability across different institutions and staining protocols. Finally, computational efficiency remains a concern, as whole-slide image processing using ViT and PatchGCN necessitates substantial computational resources, posing challenges for real-time clinical deployment. While our current hardware setup enables efficient offline analysis, the models’ inference speed and memory footprint require further optimization for point-of-care settings. To address this, future work will explore model optimization strategies—such as lightweight architecture design, model pruning, quantization, and knowledge distillation—to reduce computational overhead while maintaining robust classification performance, with the goal of achieving clinically compatible turnaround times. Additionally, the current approach has not been systematically validated across different glioma subtypes or WHO grades, which may restrict its generalizability. Further validation using larger and histologically diverse cohorts, as well as the incorporation of complementary molecular markers, will be essential to enhance the robustness and translational potential of this framework.

## Conclusions

We present FOCUS, a novel strategy that achieves state-of-the-art performance (accuracy = 0.902, AUC = 0.909) in glioma IDH mutation status classification by integrating hierarchical feature learning, cascaded feature selection, and machine learning. By leveraging a ViT for patch-level feature extraction, the model captures interpretable features reflecting nuclear morphology and tumor progression–associated protein expression. PatchGCN is employed for spatial feature aggregation, enabling the model to focus on local morphological details and capture global tumor heterogeneity. A cascaded feature selection pipeline ensures the retention of key predictive features, and a machine learning classifier is utilized for final prediction. Although statistical comparisons revealed no significant differences among the tested classifiers, the SVM offered a favorable balance of AUC, accuracy, precision and F1-score. The interpretable and reliable framework provides a valuable tool for precise classification of IDH mutation status in gliomas, with potential to enhance the efficiency of treatment decision-making and refine prognostic assessment in clinical management. 

## Supplementary Information

Supplementary Material 1.

## Data Availability

The data presented in this study are available on request from the corresponding author due to privacy and ethical reasons.

## Abbreviations

IDH: Isocitrate Dehydrogenase
FOCUS: Feature Optimization and Cascaded Unified Screening
NCAD: N-Cadherin
ViT: Vision Transformer
PatchGCN: Patch-Based Graph Convolutional Networks
SVM: Support Vector Machine
AUC: Area Under the Curve
WHO: World Health Organization
IHC: Immunohistochemical
FFPE: Formalin-Fixed, Paraffin-Embedded
ROI: Region of Interest
LASSO: Least Absolute Shrinkage and Selection Operator
MSE: Mean Squared Error
ROC: Receiver Operating characteristics
DCA: Decision Curve Analysis
CI: Confidence Interval
KNN: K-Nearest Neighbors
SGD: Stochastic Gradient Descent
XGBoost: EXtreme Gradient Boosting
LightGBM: Light Gradient Boosting Machine
TabPFN: Tabular Prior-Data Fitted Network
H&E: Hematoxylin and Eosin
