# Supplementary material for: FOCUS: a feature optimization and cascaded unified screening strategy for computer-aided pathology-based glioma molecular subtype classification
Source: Diagn Pathol. 2026 Aug 8;21:84. doi: 10.1186/s13000-026-01836-1 (PMC13733218; doi:10.1186/s13000-026-01836-1)
Supplement: Supplementary file 1 [file 13000_2026_1836_MOESM1_ESM.docx]

Supplemental Information

**FOCUS: A Feature Optimization and Cascaded Unified Screening Strategy for Computer-Aided Pathology-Based Glioma Molecular Subtype Classification**

Ya Su^a,b,c,†^, Fengqi Li^a,b,†^, Hongwei Yang^a,b^, Jie Bai^a,b^, and Jie Lu^a,b,*^

^a^Capital Medical University, Xuanwu Hospital, Department of Radiology and Nuclear Medicine, Beijing, China

^b^Capital Medical University, Beijing Key Laboratory of Magnetic Resonance Imaging and Brain Informatics, Beijing, China

^c^Beijing United Imaging Research Institute of Intelligent Imaging, Beijing, China

† Ya Su and Fengqi Li contributed equally to this work and should be considered joint first authors.

**Correspondence to:** Jie Lu, PhD. Department of Radiology and Nuclear Medicine, Xuanwu Hospital, Capital Medical University, Beijing, 100053, China; Beijing Key Laboratory of Magnetic Resonance Imaging and Brain Informatics, Capital Medical University, No. 45 Changchun Street, Xicheng District, Beijing, 100053, China. Email: imaginglu@hotmail.com.

**Detection of NCAD by Immunohistochemistry**

Formalin-fixed, paraffin-embedded (FFPE) glioma tissue blocks were sectioned into 4 μm slices using a microtome. For each case, three consecutive sections were prepared to ensure technical consistency and enable quality control. All sections underwent the same immunohistochemical (IHC) staining protocol. Following staining, the section exhibiting the most intact morphology and optimal staining quality was selected for image acquisition and subsequent computational analysis.

Immunohistochemical staining for N-cadherin (NCAD) was performed using a horseradish peroxidase (HRP)-conjugated detection system with 3,3′-diaminobenzidine (DAB) as the chromogen. The procedure included deparaffinization, rehydration, and heat-induced antigen retrieval in citrate buffer (pH 6.0) at 95–100°C for 20 minutes. After cooling, endogenous peroxidase activity was blocked using 3% hydrogen peroxide. Sections were then incubated overnight at 4°C with a primary anti-NCAD antibody, followed by a 30-minute incubation with an HRP-conjugated secondary antibody at room temperature. DAB was applied to develop a brown precipitate indicating NCAD expression in positive regions.

Following DAB staining, hematoxylin counterstaining was applied to visualize nuclei in blue-purple, providing contrast for tissue structure identification. All stained slides were independently reviewed by two experienced pathologists to assess staining quality and signal specificity. The section with the highest quality was scanned at 200× magnification using a microscope equipped with a micro-motion platform. Representative tumor regions — including the tumor core, margins, and surrounding zones — were selected based on structural integrity and staining intensity for computational analysis.

**Detection of IDH Mutation by Sanger Sequencing**

FFPE tumor tissue blocks were used for Isocitrate Dehydrogenase (IDH) mutation testing. DNA was extracted from representative tumor areas with a tumor cell content exceeding 70%, as confirmed by microscopic evaluation. Mutational hotspots in exon 4 of the IDH1 (codon 132) and IDH2 (codon 172) genes were assessed by Sanger sequencing. Polymerase Chain Reaction (PCR) amplification of the target regions was performed using gene-specific primers. The PCR products were purified and subjected to bidirectional sequencing. Sequence data were analyzed using the reference sequences (IDH1: NM_005896.3; IDH2: NM_002168.2) to identify point mutations. Only mutations confirmed in both sequencing directions were considered valid. All molecular analyses were conducted in a clinically certified diagnostic laboratory.

**Statistical Analysis Feature Screening**

T-test was performed on the initial set of 768 features to assess their ability to distinguish between IDH-mutant and IDH-wildtype gliomas. A total of 572 features showed statistically significant differences (p < 0.05) and were retained for further analysis. To reduce feature redundancy, pairwise correlation analysis was then con-ducted among the 572 features. As shown in Supplementary Figure 1, a heatmap was generated to visualize inter-feature correlations. For feature pairs with Pearson correlation coefficients greater than 0.8, only one feature from each pair was preserved. After this step, 411 features were retained for downstream modeling.

Figure S1 Heatmap of correlation matrix.

**AUC-Based Feature Screening**

To further refine the feature set, an AUC-based forward selection strategy was applied using a Random Forest classifier. As illustrated in Supplementary Figure 2, the AUC in-creased rapidly with the addition of the first few features and reached its peak when the number of features was three. Beyond this point, adding more features did not sig-nificantly improve model performance. The AUC remained relatively stable within the range of 0.680 to 0.870, suggesting that additional features introduced limited dis-criminative value.

When the number of features was fewer than or equal to three, each additional feature led to a substantial improvement in AUC, indicating their strong contribution to classification. In contrast, when the number of features exceeded three, the growth in AUC plateaued or slightly declined, reflecting diminishing marginal returns.

The final three features selected through this process were closely associated with key pathological indicators relevant to glioma subtyping.

Figure S2 The relationship between AUC and numbers of features.

**Table S1** Pairwise DeLong test results for AUC on the test set.

| Model1 | Model2 | P value |
| --- | --- | --- |
| SVM | SGD | 0.480 |
| SVM | KNN | 0.822 |
| SVM | RandomForest | 0.219 |
| SVM | XGBoost | 0.412 |
| SVM | LightGBM | 0.112 |
| SVM | TabPFN | 0.999 |
| SGD | KNN | 0.603 |
| SGD | RandomForest | 0.247 |
| SGD | XGBoost | 0.468 |
| SGD | LightGBM | 0.102 |
| SGD | TabPFN | 0.480 |
| KNN | RandomForest | 0.190 |
| KNN | XGBoost | 0.197 |
| KNN | LightGBM | 0.052 |
| KNN | TabPFN | 0.735 |
| RandomForest | XGBoost | 0.646 |
| RandomForest | LightGBM | 0.229 |
| RandomForest | TabPFN | 0.188 |
| XGBoost | LightGBM | 0.164 |
| XGBoost | TabPFN | 0.304 |
| LightGBM | TabPFN | 0.073 |
